# Laccase Gene Family in *Cerrena* sp. HYB07: Sequences, Heterologous Expression and Transcriptional Analysis

**DOI:** 10.3390/molecules21081017

**Published:** 2016-08-04

**Authors:** Jie Yang, Xinqi Xu, Tzi Bun Ng, Juan Lin, Xiuyun Ye

**Affiliations:** 1 Fujian Key Laboratory of Marine Enzyme Engineering, Fuzhou University, Fuzhou 350116, Fujian, China; T09136@fzu.edu.cn (J.Y.); xuxinqi@fzu.edu.cn (X.X.); ljuan@fzu.edu.cn (J.L.); 2 School of Biomedical Sciences, Faculty of Medicine, The Chinese University of Hong Kong, Shatin, New Territories, Hong Kong, China; b021770@mailserv.cuhk.edu.hk

**Keywords:** *Cerrena* sp., laccase, heterologous expression, qPCR, fermentation

## Abstract

Laccases are a class of multi-copper oxidases with industrial potential. In this study, eight laccases (Lac1–8) from *Cerrena* sp. strain HYB07, a white-rot fungus with high laccase yields, were analyzed. The laccases showed moderate identities to each other as well as with other fungal laccases and were predicted to have high redox potentials except for Lac6. Selected laccase isozymes were heterologously expressed in the yeast *Pichia pastoris*, and different enzymatic properties were observed. Transcription of the eight laccase genes was differentially regulated during submerged and solid state fermentation, as shown by quantitative real-time polymerase chain reaction and validated reference genes. During 6-day submerged fermentation, *Lac*7 and *2* were successively the predominantly expressed laccase gene, accounting for over 95% of all laccase transcripts. Interestingly, accompanying *Lac7* downregulation, *Lac2* transcription was drastically upregulated on days 3 and 5 to 9958-fold of the level on day 1. Consistent with high mRNA abundance, Lac2 and 7, but not other laccases, were identified in the fermentation broth by LC-MS/MS. In solid state fermentation, less dramatic differences in transcript abundance were observed, and *Lac3*, *7* and *8* were more highly expressed than other laccase genes. Elucidating the properties and expression profiles of the laccase gene family will facilitate understanding, production and commercialization of the fungal strain and its laccases.

## 1. Introduction

Laccase (benzenediol: oxygen oxidoreductase, EC 1.10.3.2) is a family of copper-containing oxidases which can oxidize a wide variety of organic and inorganic compounds. Because laccases have low substrate specificity and produce water as the by-product, they have important application values in various industrial processes, including textile refining, dye decolorization, bioremediation, lignocellulosics delignification, organic synthesis and food processing [1,2].

Laccases have been discovered in bacteria, fungi, plants and insects, and white-rot fungi are the most efficient laccase producers [1]. Fungal laccases usually exist in gene families, and the laccase isozymes display diverse expression patterns and physicochemical characteristics [3,4]. Despite the high number of publications on fungal laccases, most studies focus on one or few laccases from each organism [5,6,7]. Revealing the unique properties of each laccase isozyme will facilitate understanding their physiological roles as well as providing suitable laccases for specific application purposes [8]. Heterologous expression of individual laccase genes in hosts like *Pichia pastoris* has been widely used to characterize these industrially important enzymes although laccases are notoriously difficult to achieve active heterologous expression compared with other enzymes [8]. Laccase families in a small number of fungal species have been analyzed, among which the *Pleurotus ostreatus* laccase family has been most extensively studied. Among the 12 *P. ostreatus* laccase isozymes, six are biochemically characterized, and five are heterologously expressed in yeast [9,10,11]. In-depth transcriptional analysis showed expression patterns of *P. ostreatus* laccase genes in various strains responded to growth conditions, and Lacc2 and 10 are responsible for the majority of laccase activity [9,10]. In addition, the laccase gene families in *Auricularia auricula-judae* [12], *Coprinopsis cinerea* [13], *Flammulina velutipes* [14], *Laccaria bicolor* [15], *Lentinula edodes* [16], *Trametes hirsuta* 072 [14], *Volvariella volvacea* [17,18] have been examined and include 5–17 members.

The genus *Cerrena* is promising in laccase production and applications [19,20,21,22,23,24,25,26]. However, knowledge of *Cerrena* laccase gene families is lacking; only a handful of *Cerrena* laccase genes have been cloned and characterized. We have previously isolated *Cerrena* sp. strain HYB07 which produces laccases with high yields, stability and strong decolorizing ability [26]. Two laccases from *Cerrena* sp. HYB07, namely Lac1 and 7, have been purified from *Cerrena* fermentation broth (Lac7) or heterologously expressed in *P. pastoris* (Lac1) and characterized [26,27]. In the present study, we isolated six new members of the laccase gene family in *Cerrena* sp. HYB07 and heterologously expressed *Lac3*, *6*, *7* and *9* in *P. pastoris*. These eight laccase genes showed differential expression patterns in submerged and solid state fermentation. The work will provide a theoretical foundation for production and commercialization of *Cerrena* laccases.

## 2. Results

### 2.1. The Laccase Family of Cerrena sp. HYB07

Nine laccase genes (*Lac1*–*9*) were cloned from the genome of *Cerrena* sp. HYB07. Two of them, namely *Lac1* and *7*, have been described previously [26,27]. We were not able to amplify the cDNA of *Lac9*, and so it was excluded from subsequent analysis. Therefore, the laccase family of *Cerrena* sp. HYB07 contained at least eight members (Table 1).

The length of the nucleotide sequences for the eight laccase genes ranged from 1892 (for *Lac3*) to 2491 bp (for *Lac1*). *Lac3* had 6 introns (the fewest of the laccase gene family), *Lac6* had 10 introns, and the others had 11 each (Table 1 and Figure S1). The average size of introns for the eight laccase genes was 60 bp, with *Lac1* having the largest average intron size of 85 bp. All intron splice sites adhered to the GT..AG rule, except for the last intron of *Lac1* [27]. The introns in *Lac2*, *4* and *5* were identically positioned; so were the introns of *Lac7* and *8*.

**Figure 1:**
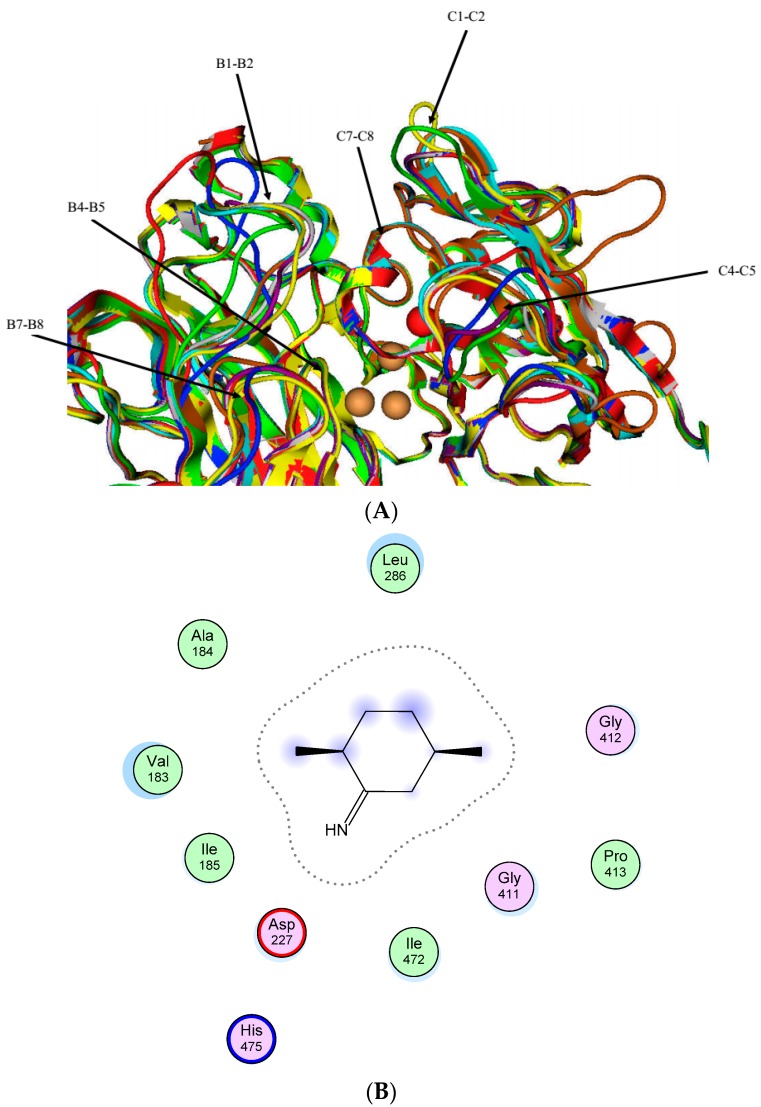
Simulated substrate binding of *Cerrena* laccases. (**A**): Superposition of the substrate-binding pockets of *Cerrena* laccases. The potential substrate-binding regions were named according to nomenclature of Hakulinen et al. [28] Red, Lac1; dark blue, Lac2; yellow, Lac3; light blue, Lac4; violet, Lac5; tan, Lac6; gray, Lac7; green, Lac8; (**B**): Interacting residues with 2,5-xylidine in Lac7 as predicted by molecular docking. Please also refer to Supplementary Table S4.

The deduced protein sequences of the eight laccases showed a length typical of fungal laccases (from 516 aa for Lac4/7 to 542 aa for Lac6) (Table 1). All the eight laccase proteins were predicted to have a signal peptide of 18–21 amino acids, the fungal laccase signature sequences L1 to L4 (Supplementary Table S3) as well as the substrate-binding loops (Figure 1 and Supplementary Table S4). Sequence identities among the eight laccases ranged from 56.6% to 76.7% (Table 2). In particular, Lac7 and 8 shared the highest identity, followed by Lac1/Lac7 and Lac2/Lac4 (>75% identities). In contrast, Lac3 and 6 showed less than 69% and 62% identities to the other family members, respectively. Compared with other HYB07 laccases, Lac6 had lower contents of α-helix and β-turn (Supplementary Table S5).

HYB07 laccases shared low to moderate identities with reported laccases. For example, Lac6 was most similar (60% identities) to laccase C from *Trametes* sp. 420 and laccase 1 precursor from *Spongipellis* sp. FERM P-18171. However, one exception did exist: the deduced Lac8 protein was 99% identical to laccase from *Cerrena* sp. CTL-2011 (GenBank No. AEL16568.1), although its identities to other laccases were lower than 80%. It is probably also worth mentioning that HYB07 laccases shared 47%–93% identities with 10 annotated laccases in the genome of *Cerrena* *unicolor* 303 (JGI Project ID: 407723) (Table 2).

### 2.2. Heterologous Expression of HYB07 Laccase Genes in P. pastoris

In an attempt to characterize individual laccase isozymes of HYB07, heterologous expression of laccase genes was carried out. In addition to previously characterized rLac1, four laccase genes, namely *3*, *6*, *7* and *8*, were heterologously expressed in *P. pastoris* (Supplementary Figure S2). Compared with BMM medium, YP was better for laccase synthesis except in the case of rLac7, and supplementary copper ions were needed in both media for higher laccase activity (Table 3). Maximum laccase yields of 0.2–6.3 U/mL were obtained after fermentation for 6–16 day. Since purification of rLac3, 7 and 8 was not successful due to their low expression levels, only purified rLac6 was characterized. rLac6 had a lower optimal pH value (3.0) than that of rLac1 (3.5), and the two laccases had identical optimal temperature (55 °C).

### 2.3. Quantification of Laccase Transcripts with qPCR

Expression stability of seven housekeeping genes during submerged and solid state fermentation was evaluated by geNorm (Table S6). The two most stable genes were *18S rRNA* and *Cyt-c* for submerged fermentation and *β*-*tubulin* and *EF1-α* for solid state fermentation. These two gene pairs were used as the references for subsequent laccase gene transcription profiling experiments.

**Figure 2:**
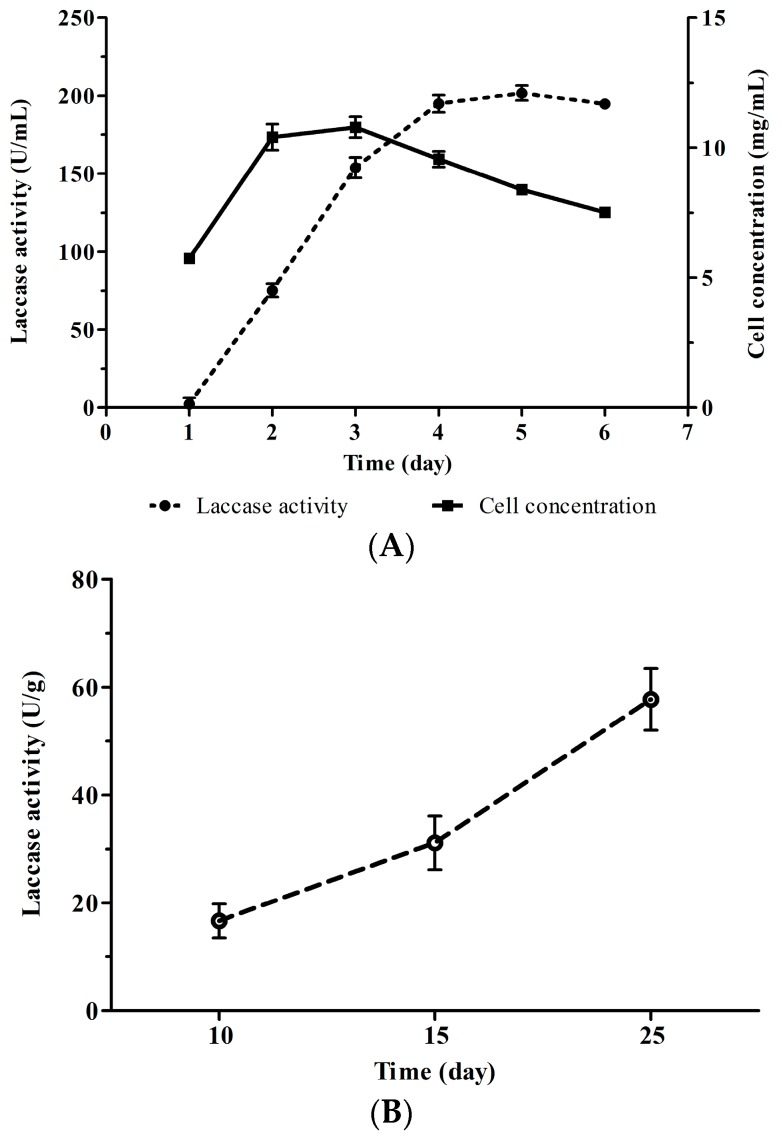
Laccase production of *Cerrena* sp. HYB07 in submerged (**A**) and solid state fermentation (**B**).

**Figure 3:**
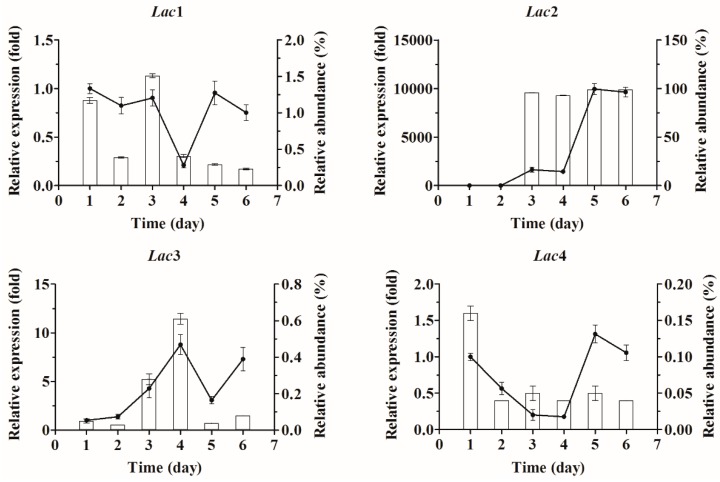

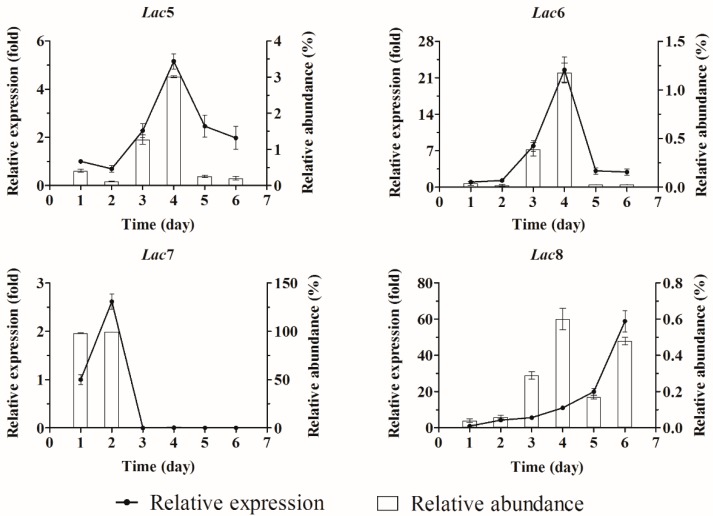
Relative expression of the eight laccase genes in *Cerrena* sp. HYB07 during submerged fermentation. Transcript levels of each gene on days 1–6 are expressed as fold changes compared to the transcript level on day 1. The reference genes used for normalization were *18S rRNA* and *Cyt-c*. Relative transcript abundance (expressed as a percentage) of each laccase gene compared with all laccase transcripts on days 1–6 was also shown.

Since extracellular laccase activity and biomass of *Cerrena* sp. HYB07 in liquid fermentation were highest on days 4–5 and day 2, respectively (Figure 2A), transcription of the eight laccase genes was examined for a period of 6 days, and differential expression patterns were observed (Figure 3). For each laccase gene, the transcript level on each day was compared to the level on day 1 and expressed as a fold change (relative expression); the transcript level was also compared with the total transcript level of the laccase gene family and expressed as a percentage (relative abundance). *Lac7* was predominantly expressed on the first two days, accounting for over 98% of all laccase transcripts. On day 3, *Lac7* transcription level plummeted to 0.2% of the level on day 1 and remained low. Interestingly, accompanying *Lac7* downregulation, *Lac2* transcription was drastically upregulated by 1649-fold on day 3 (becoming the major laccase gene expressed), followed by another upregulation on day 5 to 9958-fold of the level on day 1. Transcription patterns of *Lac5* and *6* were similar; their expression was first upregulated, peaked on day 4 (5- and 23-fold of the respective day-1 levels), and was then downregulated to approximately two-fold on day 6. In contrast, *Lac1* and *4* transcription was lowest on day 4 and then returned to similar levels as day 1. Similar to *Lac5* and *6*, *Lac3* also displayed the highest transcription level on day 4, which was followed by a drop on day 5. Contrary to *Lac5* and *6*, expression of *Lac3* was upregulated again on day 6 to a level similar to that on day 4. *Lac8* expression continued to increase during the fermentation and reached the maximum on day 6 to 59-fold of the level on day 1.

Considering the dramatic changes in *Lac2* and *7* transcription patterns, LC-MS/MS was adopted to identify extracellular laccases on day 5 (Table 4). Only Lac2 and 7 were observed in the fermentation broth based on the presence of unique peptides (at least two per protein). On the other hand, although one Lac6 peptide was found, its presence could not be confirmed by a second unique peptide.

**Figure 4:**
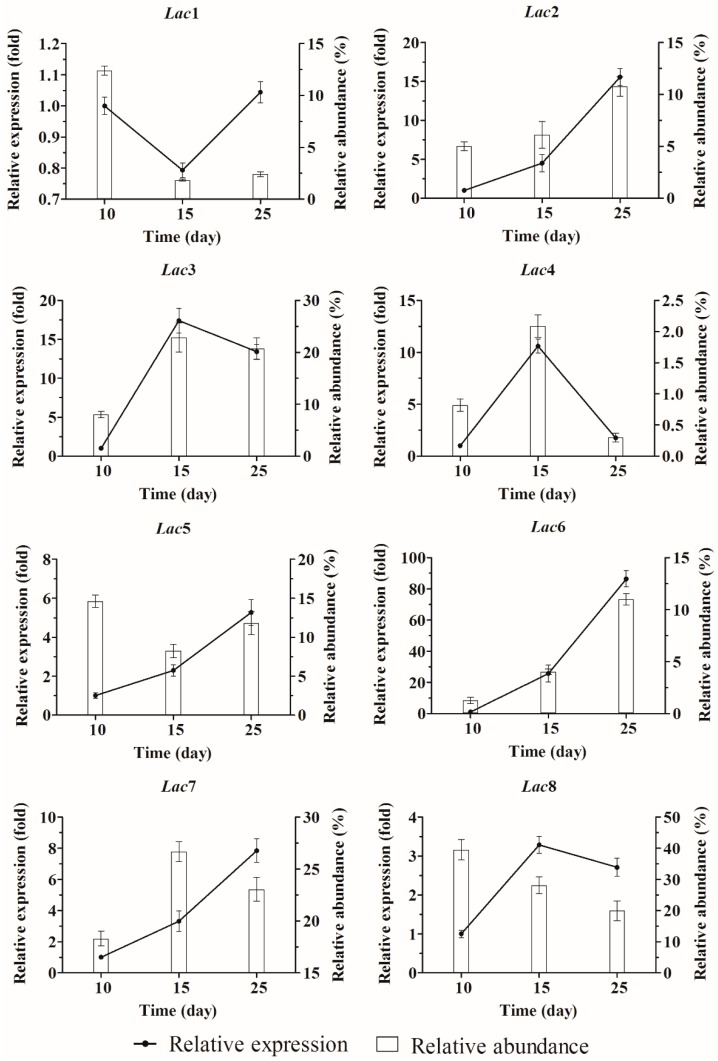
Relative expression of the eight laccase genes in *Cerrena* sp. HYB07 during solid state fermentation. Transcript levels of each gene on days 10, 15 and 25 are expressed as fold changes compared to the transcript level on day 10. The reference genes used for normalization were *β*-*tubulin* and *EF1-α*. Relative transcript abundance (expressed as a percentage) of each laccase gene compared with all laccase transcripts on days 10, 15 and 25 was also shown.

Extracellular laccase activity increased during solid state fermentation (Figure 2B). qPCR analysis revealed that unlike in submerged fermentation, no laccase gene was predominantly expressed in solid state fermentation (Figure 4). However, *Lac3*, *7* and *8* mRNAs were more abundant than the others, and *Lac4* was the least expressed. Expression of *Lac2*, *5*, *6* and *7* increased with time, whereas expression of *Lac1* remained constant. *Lac3*, *4* and *8* transcription levels were highest on day 15, and transcription of *Lac4*, but not *Lac3* or *8*, was downregulated significantly after day 15.

## 3. Discussion

The *Cerrena* sp. HYB07 laccase family contained at least eight members sharing moderate identities to each other as well as other published laccases. These laccases belonged to classes 2 and 3, depending on the amino acid located 10 amino acids downstream of the conserved Cys in the L4 domain [29]. Specifically, Lac1, 3, 4 and 7 were class 2 (Leu) laccases, whereas Lac2, 5, 6 and 8 were class 3 (Phe) laccases. Even though laccases harboring Phe and Leu had been correlated with high and medium redox potentials (E^0^), respectively [29], it was later shown that Phe at this position may not be a prerequisite for a high E^0^ [30]. Instead, a glutamic acid corresponding to Glu-460 and a serine residue and corresponding to Ser-113 in TvL (*T. versicolor* laccase with a high E^0^ of 800 mV) are thought to be more important for a high E^0^ and are also conserved in other high E^0^ enzymes [31]. Since these two amino acids were found in all HYB07 laccases except Lac6, we speculated that HYB07 laccases had high redox potentials. This was unlike the laccase families from *L. bicolor* or *V. volvacea*; all laccases from these two species are predicted to have medium redox potentials [15,18].

In opposite to the highly conserved fungal laccase signature domains, more sequence variations with respect to length and amino acid composition, existed in the potential substrate-binding loops, suggesting the laccase isozymes might have different substrate ranges and catalytic properties [32]. Molecular docking revealed putative amino acids in Lac7 that interacts with 2,5-xylidine, many of which correspond to those in contact with the substrate in LacIIIb in *T. veriscolor* [33], such as Val 183, Ile 185, Asp227, Leu286 and His 475.

Since isolation of laccases with low expression levels from the fermentation broth of *Cerrena* sp. HYB07 was not possible, heterologous expression with *P. pastoris* as the host was adopted in order to characterize the individual laccases. Five laccase genes, namely *Lac1*, *3*, *6*, *7* and *8*, were expressed under the AOX1 promoter and α-factor signal peptide, and recombinant laccases were successively secreted. *P. pastoris* is a popular host for heterologous laccase production [34], although laccase yields by *P. pastoris* typically do not exceed 10 U/mL and often require a long production cycle (e.g., ≥10 day) [8,35,36], which might at least partially be accounted for by difficult expression of laccases in *P. pastoris*. Indeed, expression levels of the recombinant *Cerrena* laccases in our hands were within the range of reported laccases, although much lower than that of the native host, especially in the case of *Lac7*, a predominantly expressed laccase gene of *Cerrena* sp. HYB07 (compare the rLac7 yield of 0.5 U/mL after 10-day fermentation of *P. pastoris* with laccase yield of 75 U/mL after 2-day fermentation of HYB07). On the other hand, rLac6 required a production cycle longer than other recombinant HYB07 laccases, but close to the production cycles of *Coprinus comatus* [8] and *Ganoderma* [35] laccases expressed in *P. pastoris*. Codon optimization of *Lac6* combined with the native signal peptide only moderately increased laccase yields (by approximately two-fold) and did not shorten the production cycle (data not shown). Therefore, we concluded that *P. pastoris* might not be desirable for laccase expression, and no further work was carried out to improve laccase yields of recombinant *P. pastoris*. Nonetheless, characterization of recombinant laccases not only facilitates our understanding of individual isozymes, but also allows protein engineering [34].

HYB07 laccase (as a mixture) was most reactive at pH 3.0 and 30 °C. Consistently, rLac6 and Lac7 (previously purified from HYB07 fermentation broth), but not rLac1, showed maximum activity at 3.0. Large discrepancies were observed with respect to optimal temperatures. The temperature optima (45–55 °C) of purified rLac1, Lac7 and rLac6 isozymes were all higher than 30 °C, which was the optimal temperature of HYB07 laccase.

Throughout fermentation, complex and distinct expression patterns of laccase genes were observed. *Lac1* expression levels showed little fluctuations and were the most stable of the family. Transcription levels of most other laccase genes were higher at later growth phases compared to earlier phases of fermentation, except for *Lac7* in submerged fermentation. The most highly expressed genes differed in submerged and solid state fermentation. The dramatic suppression of *Lac7* and concomitant upregulation of *Lac2* in the liquid culture might be accounted for by depletion of nutrients, aging of cells, as well as accumulated oxidative stresses [37]. More work is needed to elucidate the molecular mechanisms underlying regulation of laccase gene transcription. Although mRNA abundance does not always correlate with protein abundance, Lac2 and 7 were indeed identified in HYB07 fermentation broth by LC-MS/MS analysis, confirming that these two laccases were the main sources of laccase activity in the liquid culture, as anticipated from qPCR results. Presence of the other laccases were either not detected or confirmed, presumably caused by their low transcription levels. Lcc1 and Lcc5 were identified as the main laccases secreted in liquid cultures of *Coprinopsis cinerea* strains by LC-MS/MS analysis [38]. Predominance of certain laccases was also reminiscent of the case of *P. ostreatus*: Lacc2 and 10 are responsible for most of laccase activity in submerged cultures, although successive high-level expression of these two laccase genes was not observed [9].

Submerged fermentation of *Cerrena* sp. HYB07, with a shorter production cycle and higher enzyme yields, was more suitable for laccase production compared with solid state fermentation. Expression patterns of the laccase genes were dissimilar in submerged and solid state fermentation, probably due to different growth media and conditions. Similarly, *Lentinula edodes* laccase transcripts were observed at different levels in mycelia in liquid and sawdust fermentation [16], One of the many differences was the available carbon source: glucose in the PDY medium is easily utilized, while the solid medium contained mostly lignocellulose in the form of sawdust. Furthermore, HYB07 laccase production did not require an inducer such as sawdust, unlike *Trametes* *versicolor* HEMIM-9 [32]. The distinct transcription responses of laccase genes reinforced that the laccase isozymes had different physiological roles and were subject to differential expression regulation.

In short, the laccase family in *Cerrena* sp. HYB07, a laccase-producing strain with industrial potential, consisted of at least eight genes. The laccase isozymes displayed divergent enzymatic properties and transcription profiles. Future work is warranted to further understanding of the biochemistry, expression regulation and function of *Cerrena* HYB07 laccases. The research findings will pave the way for commercialization and applications of the fungal strain as well as its laccases.

## 4. Materials and Methods

### 4.1. Fermentation of Cerrena sp. HYB07

*Cerrena* sp. strain HYB07 [26] was preserved in the culture collection of the College of Biological Sciences and Technology at Fuzhou University, Fuzhou, China and maintained on potato dextrose agar (PDA) (Difco, Leeuwarden, The Netherlands) at 4 °C. For laccase fermentation, 5 mycelial plugs (1 cm diameter) were removed from the peripheral region of a 4-day-old PDA plate and inoculated in 50 mL potato dextrose broth (PDB) seed medium. After growing for 2 day at 30 °C with a shaking speed of 200 rpm, an aliquot (8% of the final volume) was transferred to a second PDB medium. After another 2 days, the second seed culture was used to inoculate PDY fermentation medium at the final concentration of 8% (*v*/*v*). Submerged fermentation was carried out at 30 °C and 200 rpm. PDY was PDB medium supplemented with 0.5% yeast extract and 0.25 mM CuSO_4_. Samples were collected every 24 h for enzyme activity assays, biomass measurements and RNA extraction.

Solid state fermentation was carried out in triplicate in glass jars filled with 105 g medium (78% broad-leaved sawdust, 20% wheat bran, 1% lime, 1% plaster) with 65% water content. Sawdust was kindly provided by Fujian Fuquanxin Biological Technology Co. Ltd. (Ningde, Fujian, China). The jars were autoclaved for 3 h at 121 °C. Each jar was inoculated with seven mycelial plugs (1 cm diameter) and stored at 28 °C. Samples were harvested on days 10, 15 and 25 for enzyme activity assays, biomass measurements and RNA extraction. Enzyme was extracted with double distilled water (solid/solution ratio 1:10) at 30 °C and 120 rpm for 2 h, and the supernatant was collected by centrifuging at 8000 *g* for 15 min. On day 10, the strain colonized half of the medium; on day 15, the strain fully colonized the medium. We were not able to obtain fructification in two months at 90% relative humidity with low temperature (15 °C) and/or daylight.

### 4.2. Enzyme Activity Assay

Laccase activity was assayed according to a previously published method [39] with slight modifications. Laccase activity was assayed at 30 °C and pH 3.0 with 0.25 mM ABTS (ε = 36,000 M^−1^·cm^−1^) as the substrate by following absorbance changes at 420 nm for 5 min unless otherwise stated. One unit of enzyme activity (U) was defined as the amount of laccase required to oxidize 1 μmol ABTS per min. All assays were carried out in triplicate.

### 4.3. Nucleic Acid Extraction

For submerged fermentation, mycelia were harvested from the liquid culture by pressure filtration. For solid state fermentation, mycelia were separated from solid medium. Mycelia were frozen in liquid nitrogen before storage at −80 °C before nucleic acid extraction. Genomic DNA was isolated with the DNAquick Plant System (TIANGEN, Beijing, China) according to the manufacturer’s instructions.

For RNA extraction, mycelia from three biological replicates were pooled, and total RNA was extracted with Trizol (Thermo Fisher Scientific, Waltham, MA, USA). RNA quantity was measured at 260 nm with a UV-Vis spectrophotometer (U-2910, HITACHI, Tokyo, Japan). For all RNA preparations, the ratios of the absorbance at 260 and 280 nm were between 1.80 and 1.95. RNA integrity was checked by 1% agarose gel electrophoresis before reverse transcription. Contaminating genomic DNA was removed with RNase-Free DNase (Promega, Madison, WI, USA), and reverse transcription was conducted by using the RevertAid First Strand cDNA Synthesis Kit (Fermentas, ON, Canada) and 2 μg total RNA.

### 4.4. Cloning of Laccase Genes

Laccase fragments covering the four copper-binding motifs of fungal laccases (L1–L4) were amplified with the degenerate primers L-1F and L-4R (Supplementary Table S1) [40] with HYB07 genomic DNA as the template. PCR products were inserted into pMD18-T vector (Takara, Kusatsu, Shiga, Japan) and transformed into *E. coli* DH5α competent cells. Two hundred independent positive clones were subjected to diagnostic restriction enzyme digestion patterns to identify different fragments. Four positive clones of each fragment were sequenced. Thermal asymmetric interlaced-polymerase chain reaction (TAIL-PCR) [41,42] was used to amplify the 5’ and 3’-flanking sequences of the novel laccase fragments (Supplementary Table S1).

Based on complete gene sequences, primers for RT-PCR were designed to include the start and stop codons, respectively (Supplementary Table S1). Laccase cDNAs were amplified with Ex Taq DNA polymerase (Takara).

### 4.5. Bioinformatics Analysis

The laccase sequences were analyzed using Vector NTI (Life Technologies) and BLAST [43]. Signal peptide was predicted with SignalP 4.1 (http://www.cbs.dtu.dk/services/SignalP/) [44]. The theoretical isoelectric point (pI) and molecular weight (MW) were calculated by using the Compute pI/Mw tool (http://web.expasy.org/compute_pi/) [45]. Potential *N*-glycosylation sites (Asn-X-Ser/Thr) were identified with NetNGlyc 1.0 Server N (http://www.cbs.dtu.dk/services/NetNGlyc/). Alignments of laccase proteins and the identity table were generated with Clustal Omega (http://www.ebi.ac.uk/Tools/msa/clustalo/) [46]. Homology modeling and molecular docking were performed with Swiss-Modeling Workspace Version 8.05 [47] and Molecular Operating Environment (Chemical Computing Group Inc., Montreal, QC, Canada). Secondary structures were predicted with SOPMA Secondary Structure Prediction Method [48].

### 4.6. Heterologous Expression of Laccase Genes in Pichia pastoris

Laccase gene coding sequences without the predicted signal sequences were amplified (Supplementary Table S1) and inserted into the yeast expression vector pPICZα C (Life Technologies). *P. pastoris* X33 cells (Life Technologies) were transformed with a Gene Pulser II apparatus (BioRad, Richmond, CA, USA) as described in the *Pichia* Expression Kit User Manual. Positive transformants (X33/pPICZα-Lac) were inoculated in YPD medium at 28 °C with shaking at 200 rpm. After 24 h, cells were harvested by centrifugation and resuspended to OD_600_ of 2.0 with YP or BMM medium (pH 6.0) supplemented with 0.4–1 mM Cu^2+^ ions. Methanol at 1% (*v*/*v*) was added every 24 h, and extracellular laccase activity was recorded every day. For each heterologously expressed laccase gene, over 50 positive transformants were screened, and the one with highest laccase production level was selected for subsequent analysis. The strain transformed with the original pPICZα vector (X33/pPICZα) was prepared as the negative control and analyzed in parallel.

### 4.7. Enzymatic Properties of Recombinant Laccases

Fermentation broth was precipitated with ammonium sulfate, and laccase purification was carried out with anion exchange chromatography as previously described [27]. ABTS was the substrate for all assays.

To determine the optimum pH, laccase activity was measured between pH 2.2 to 7.0 in Na_2_HPO_4_-citric acid buffers; the relative enzyme activity was calculated with the highest activity as 100%. For the optimum temperature, laccase activity was measured at the optimum pH and various temperatures ranging from 25 to 75 °C. All experiments were performed in triplicate.

### 4.8. Quantitative Real-Time Polymerase Chain Reaction (qPCR)

qPCR primers (Supplementary Table S2) were designed by PrimerQuest (Integrated DNA Technologies, Coralville, IA, USA). The amplicon sizes ranged from 91 to 116 bp with the exception of the *β-tubulin* amplicon (220 bp). The amplification efficiencies of the primers were between 91.2% and 100.7% (R^2^ ≥ 0.99).

qPCR was performed on an Applied BioSystems 7500 Real-Time PCR system (Life Technologies). Each reaction mixture (20 μL) contained 10 μL 2 × GoTaq qPCR Master Mix (Promega), forward and reverse primers (each at 200 nM) and 2 μL cDNA (1:50 diluted). Amplification was carried out as follows: an initial denaturation step of 95 °C for 2 min, followed by 40 cycles of 95 °C for 15 s and 60 °C for 1 min. A dissociation step was added after qPCR cycles to check amplification specificity. All reactions were performed in triplicate. RT negative controls (containing RNA template without reverse transcription) were run for every sample by using *18S rRNA* as the target to check for DNA presence. No-template negative controls were included every run to detect possible contamination or carryover. Relative quantities of the laccase transcripts were calculated by using ExpressionSuite v1.0.3 (Life Technologies) with the comparative Ct method [49].

### 4.9. Reference Gene Validation

Expression stability of seven housekeeping genes, including *18S rRNA*, *ATP6* (ATP synthase 6), *Cyt-c* (cytochrome c oxidase subunit 1), *EF1-α* (translation elongation factor 1-alpha), *GAPDH* (glyceraldehyde 3-phosphate dehydrogenase), *RPB2* (RNA polymerase II second largest subunit) and *β*-*tubulin*, in *Cerrena* sp. HYB07 was examined. Samples were divided into two groups for data analysis. Group A consisted of six samples from submerged fermentation, and Group B consisted of three samples from solid state fermentation. The two most stable housekeeping genes evaluated by geNorm [50] were used as the reference genes for subsequent qPCR analysis.

### 4.10. LC-MS/MS Analysis of Secreted Laccases

The fermentation broth of a 5-day-old culture was collected, and extracellular proteins were separated by SDS-PAGE and stained with Coomassie Brilliant Blue R-250. The protein band corresponding to laccases was excised and submitted to LC-MS/MS analysis by Shanghai Applied Protein Technology Co., Ltd. (Shanghai, China). Protein digestion was performed according to the FASP procedure as previously described [51]. Proteins were digested with 2 μg trypsin (Promega) in 40 μL 25 mM NH_4_HCO_3_ overnight at 37 °C.

The fractionated digests were analyzed by online C_18_ reversed-phase micro-scale liquid chromatography tandem mass spectrometry. The Ettan MDLC (GE Healthcare, Pittsburgh, PA, USA) equipped with an Empore SPE Cartridge (Sigma-Aldrich, St. Louis, MO, USA) was used for desalting and separation of peptides prior to online LTQ Velos (Thermo Fisher Scientific) analysis. Each scan cycle consisted of one full scan mass spectrum (*m*/*z* 300–1800) followed by 20 MS/MS events of the most intense ions with the following dynamic exclusion settings: repeat count 2, repeat duration 30 s, exclusion duration 90 s. The samples were loaded onto trap column firstly with 10 μL/min flow rate, and then the desalted samples were eluted at a flow rate of 1200 nL/min in MDLC by applying a linear gradient of 0%–50% B for 60 min. The LTQ linear ion trap mass spectrometer was used for MS/MS experiment with ion transfer capillary of 160 °C and ISpary voltage of 3 kV. Normalized collision energy was 35.

All .dta files were created by using Bioworks Browser rev. 3.1 (Thermo Fisher Scientific) with precursor mass tolerance of 1.4 Da, threshold of 100, and minimum ion count of 10. The acquired MS/MS spectra were searched against the concatenated target/reverse database containing the eight *Cerrena* laccase sequences by using the SEQUEST search engine. Searches were performed in the trypsin enzyme parameter in the software. Methionine oxidation was only specified as a differential modification and cystine carbamidomethylation was a fixed one. Two missed cleavages were allowed. All output results were combined by using in-house software called buildsummary [52]. The filter was set to FDR ≤ 0.01.

## Figures and Tables

**Table 1:** The eight laccase genes cloned from *Cerrena* sp. HYB07.

Laccase Gene	GenBank #	DNA Length (bp) ^a^	Intron #	Mature Protein (aa)	Signal Peptide (aa)	MW (kD)	pI	Glycosylation Sites
*Lac1*	KC540913	2491	11	497	20	54.1	5.5	1
*Lac2*	KF317944	2212	11	505	21	54.7	5.2	1
*Lac3*	KF317945	1892	6	497	21	53.1	4.4	5
*Lac4*	KF317946	2170	11	496	20	53.6	5.4	0
*Lac5*	KF317947	2199	11	504	21	55.4	5.3	1
*Lac6*	KF317943	2179	10	524	18	57.9	5.5	6
*Lac7*	KF317949	2168	11	495	21	52.9	5.6	1
*Lac8*	KF317948	2181	11	498	21	53.7	5.0	4

^a^ From ATG to the stop codon. #, number.

**Table 2:** Amino acid sequence identities (%) of deduced laccases of *Cerrena* sp. HYB07 and *C. unicolor* 303.

Laccase	Lac1	Lac2	Lac3	Lac4	Lac5	Lac6	Lac7	Lac8	193382	310741	357631	364416	383410	390832	390880	395909	408157
Lac1		69.5	66.0	69.3	65.4	60.3	76.3	71.6	71.1	72.4	63.8	65.8	69.4	67.8	52.7	85.1	65.7
Lac2			63.8	75.3	72.5	61.5	73.0	67.3	68.4	69.8	62.0	88.4	64.8	74.4	62.5	68.2	73.8
Lac3				63.7	58.5	56.6	68.5	66.7	66.5	63.3	88.8	61.5	61.7	63.0	46.9	64.9	58.4
Lac4					68.9	60.9	72.2	67.8	69.2	71.4	62.8	72.3	67.6	92.1	57.0	69.7	68.1
Lac5						59.8	68.6	64.6	64.1	65.1	57.8	71.2	61.9	67.3	59.0	63.8	92.8
Lac6							60.4	58.9	55.8	56.5	53.7	60.0	54.5	56.7	47.0	55.1	57.9
Lac7								76.7	82.5	78.1	66.5	69.6	75.2	72.2	54.0	73.8	68.1
Lac8									73.2	71.5	64.5	64.5	70.6	67.2	50.9	68.8	65.0
193382										75.8	64.5	68.2	71.7	69.9	50.6	71.7	65.3
310741											62.3	68.3	73.9	71.2	54.0	72.8	66.3
357631												60.4	61.7	63.9	46.5	63.6	58.6
364416													63.8	74.1	61.9	66.5	72.5
383410														66.0	51.5	69.0	63.3
390832															57.8	69.9	67.9
390880																53.3	60.6
395909																	65.2
408157																	

Lac1–8 are laccases from *Cerrena* sp. HYB07 described in this study. Laccases from *C. unicolor* 303 (JGI Project ID: 407723) are designated by the protein IDs.

**Table 3:** Enzymatic properties of *Cerrena* laccase isozymes heterologously expressed in *P. pastoris*.

Laccase	Laccase Yield (U/mL)	Medium	Cu^2+^ Concentration	Production Cycle (days)	Optimum pH	Optimum Temp (°C)	Reference
rLac1	6.3	YP	0.4	9	3.5	55	[27]
rLac3	0.2	YP	0.5	6	NA	NA	This study
rLac6	2.9	YP	1.0	16	3.0	55	This study
rLac7	0.5	BMM	0.4	10	NA	NA	This study
rLac8	0.7	YP	0.5	7	NA	NA	This study

NA, not available. Purification of rLac3, 7 and 8 was not successful due to their low expression levels, and thus their characterization was not carried out. ABTS was used as the substrate. YP, yeast extract peptone medium. BMM, buffered minimal methanol medium. Medium recipes can be found in the *Pichia* Expression Kit User Manual.

**Table 4:** LC-MS/MS analysis of extracellular laccases in the fermentation broth of *Cerrena* sp. HYB07 on day 5.

Laccase	Score	Coverage	Unique Peptides	Total Peptides	PSMs
Lac2	66.67	27.72	9	9	22
Lac6	2.55	2.86	1	1	1
Lac7	676.22	41.82	13	13	205

Score, statistic reliability of protein identification; coverage, percentage of tryptic peptides identified; PSM, peptide spectrum matches.

## Supplementary Materials

Supplementary materials can be accessed at: http://www.mdpi.com/1420-3049/21/8/1017/s1.
