# Supplementary material for: Laccase Gene Family in Cerrena sp. HYB07: Sequences, Heterologous Expression and Transcriptional Analysis
Source: Molecules. 2016 Aug 4;21(8):1017. doi: 10.3390/molecules21081017 (PMC6273318; doi:10.3390/molecules21081017)
Supplement: Supplementary file 1 [file molecules-21-01017-s001.pdf]

Supplementary Materials: Laccase Gene Family in *Cerrena* sp. HYB07: Sequences, Heterologous Expression and Transcriptional Analysis

Jie Yang, Xinqi Xu, Tzi Bun Ng, Juan Lin and Xiuyun Ye

Table S1. Primers used to clone laccase genes from *Cerrena* sp. HYB07.

Gene	Primer Sequence	Purpose
Laccase L1-L4 fragment	L-1F: CAYTGGCAYGGNTTYTTYCA	Degenerate PCR [1]
	L-4R: TGVHARTCDATRTGRCARTG	
	R = A/G, Y = C/T, M = A/C, K = G/T, S = G/C, W = A/T, H = A/T/C, D = G/A/T, B = G/T/C, V = G/A/C, N = A/T/G/C.	
*Lac*2	Lac2-SP1: AATGTAGTTAGGGTCGCAGGAGATAGAGA	TAIL-PCR amplification of 5′-flanking sequence
	Lac2-SP2: GTCGTAGAGGTGCTTGTGTGGATCGTTAGG	
	Lac2-SP3: AAGAGTTTCCAGTGACAATAGGGCAT	TAIL-PCR amplification of 3′-flanking sequence
	Lac2-SP4: CATCCCTTCCATTTGCACGGTGTAAGTCC	
	Lac2-SP5: ATCTTACCTCGTTTCTTACTCACCATGCC	
	Lac2-SP6: TTCAATGACGCTATCCTCCGA	Amplification of cDNA
	Lac2-RT-1: ATGCCCTCTAACTTCCTTTCATTGAC	
	Lac2-RT-2: TTGGTTTAGATGGCGGTACCCTTC	
*Lac*3	Lac3-SP1: CCAGTCAAGCAAGGGTAATAACCGTG	TAIL-PCR amplification of 5′-flanking sequence
	Lac3-SP2: TCGGAGAACCATCGCAATACTGA	
	Lac3-SP3: AAGAGTTTCCAGTTGCGATTG	TAIL-PCR amplification of 3′-flanking sequence
	Lac3-SP4: TTCCTCCTATCGTCCCTGTTCTCCTCCAAA	
	Lac3-SP5: TCCTCCCATCCGGCAGCGTCTTCTCATT	
	Lac3-SP6: GCTCCGACACCTACAACTACGCCAACC	Amplification of cDNA
	Lac3-RT-1: GCGGATGTTTAACTTTAATTCTTTGCTC	
	Lac3-RT-2: CTTGTCAGAGTCAGCGAGGGC	
	Lac3E-1: GAATTCAGCAATCGGTCCTGTCACTGA (*Eco*RI)	Construction of expression vector
	Lac3E-2: TCTAGATTACTTGTCAGAGTCAGCGAGGGC (*Xba*I)	
*Lac*4	Lac4-SP1: TCACACCCGAAACCTACCATCATCAACATC	TAIL-PCR amplification of 5′-flanking sequence
	Lac4-SP2: ATCATAGACGACAAAGGCTCCCCGAAG	
	Lac4-SP3: AGACCATCGCAGTATTGAGTAGACA	
	Lac4-SP4: GCTTCCTTCACACCTCCCACAGTCCCG	
	Lac4-SP5: TTGGAGCAGAATAAGGTGGTGGAGAT	TAIL-PCR amplification of 3′-flanking sequence
	Lac4-SP6: ATGAATGCTCCTATTCGTGATGTTGTTA	Amplification of cDNA
	Lac4-RT-1: TAAACCCACCTGAGAGCGATAACGATGT	
	Lac4-RT-2: TATTATGTATCTCCTGGGCTCAAG	
*Lac*5	Lac5-SP1: CCATTGATAAGAGTCGAGTCCGGTGTCCTG	TAIL-PCR amplification of 5′-flanking sequence
	Lac5-SP2: GAGGGTCTTCCGGGTCATACACGA	
	Lac5-SP3: AGTATTGGGTAGACAGGTGGCT	TAIL-PCR amplification of 3′-flanking sequence
	Lac5-SP4: CATCCTTTCCATCTCCACGGTGTCAGTCAT	
	Lac5-SP5: ATTCTTACAGCACAACTTCTGGGTCGT	
	Lac5-SP6: CTATCCGTTTCGTGGTATGACTTCC	Amplification of cDNA
	Lac5-RT-1: CTCTCCAAGCGATGGTGTCAAA	
	Lac5-RT-2: CCGCTTAATTGGTCGTACTGTTGTTG	

Table S1. *Cont.*

Gene	Primer Sequence	Purpose
*Lac*6	Lac6-SP1: CCGTTAATCAAGGTAGAGTCAGGTGTA<br>Lac6-SP2: AGCAGCACTTACTATCGTCTATGTCGT	TAIL-PCR amplification of 5′-flanking sequence
	Lac6-SP3: CAGTATTGTGTGCGTGAGTGAC<br>Lac6-SP4: GACCTCGTTATTCCTCCCCTCAAGAT	TAIL-PCR amplification of 3′-flanking sequence
	Lac6-SP5: TACACCCATTCTGACCGCTATTC<br>Lac6-SP6: TTCTGGGTCATTCAAAGTGC<br>Lac6-RT-1: ATGGTGCCCGCGCTATCCC<br>Lac6-RT-2: TCGGATTCGCTAAACCAAGACTA	Amplification of cDNA
	Lac6E-1: GCAGGAATTCATTTACCACAGAGCCGCTTTC (*Eco*RI)<br>Lac6E-2: CGGCCC*TCTAGA*CTATGGAACTAAGTTGTCGT (*Xba*I)	Construction of expression vector
Lac7	Lac7E-1: TTACTCGAGAAAAGAGAGTGAAGCTGCGCC GTTGGTCCTGTCAC (*Xho*I)<br>Lac7E-2: GGGCGGTCTAGATTACTTGTCACCATCAGCA (*Xba*I)	Construction of expression vector
*Lac*8	Lac8-SP1: CGCCTCGTAGACCGTCGCA<br>Lac8-SP2: GGGATAGGGCACTGGTTCACGA	TAIL-PCR amplification of 5′-flanking sequence
	Lac8-SP3: AAGAAGCCATGCCAGTGAA<br>Lac8-SP4: CGATTTCTGGACATACTCTTGGTTTCT	TAIL-PCR amplification of 3′-flanking sequence
	Lac8-SP5: CTCTATCTAACGCAGCCTCACTAAAG<br>Lac8-SP6: ACTGTAGCATAACTTCCACG<br>Lac8-RT-1: GATGTCTTTTCGCGCTGCA<br>Lac8-RT-2: TTTGTCGCCTTCGGGCAGA<br>Lac8E-1: GAATTCAGCCATTGGCCCCGTCGC (*Eco*RI)<br>Lac8E-2: GCGGCCGCCTAGAGATTTTTGTCGCCTT (*Not*I)	Amplification of cDNA<br>Construction of expression vector

Arbitrary degenerate primers for TAIL-PCR used were same as previously described [2].

Table S2. qPCR primers used in this study.

Primer	Sequence
Lac1-F	CTTGGTTCCTCCACTGTCATATC
Lac1-R	GTTATTCCAGGACTCAGGAACAG
Lac2-F	GGCCAAACTGGTTACAATTTCA
Lac2-R	GAACCAAGGTCCAGGGTTATC
Lac3-F	CACATCGACTGGCATTTGGA
Lac3-R	GTCAGCAGGGATGTTAGTGTTAG
Lac4-F	CGGGCAAACCACATACAACTA
Lac4-R	CCGGGATTATCGGTCACAAATC
Lac5-F	ACATTGACTGGCACTTGGA
Lac5-R	CAGTCCTTAGGTGTTGGGTTAG
Lac6-F	CGTTAGGGACGTGGTGAATATC
Lac6-R	CGATATGGCAGTGGAGGAAC
Lac7-F	CTGGTCAAACTACTCCCAACTAC
Lac7-R	GGTGGTGAAACGGATGGTAA
Lac8-F	CAGGAGAGACCACCTACAATTATG
Lac8-R	GTTGTCAGTAGTGAAGCGGATAG
18S rRNA-F	AGACGGAAGTTTGAGGCAATAA
18S rRNA-R	CTTCCGGCCAAGGTGAATAA
ATP6-F	CAAGAGCTAATGGAGTACCTGAA
ATP6-R	CACTATATGGACGGCTGTTACT
Cyt-c-F	CTGATATGGCCTTCCCTAGATTG
Cyt-c-R	CATCCTGTACCAGCTCCATTT
EF1-F	CTACCAACGTGACCACTGAA
EF1-R	GACGTTCTTGACGTTGAAACC
β-tubulin-F	TTAGGTCGCCACTATCTTCCG
β-tubulin-R	AACTGGTCGCTGACACGCT
GAPDH-F	CCGAGTACTTGGAGTCGTATTG
GAPDH-R	TGCCAAGAAGGTCATCATCTC
RPB2-F	GTATGGTTTGTCCTGCTGAAAC
RPB2-R	GAGAACGAACCGACGGAAATA

Table S3. Alignment of the signature sequences (L1-L4) of *Cerrena* sp. HYB07 laccases.

Protein	L1	L2	L3	L4
Lac1	HWHGFFQKGTNWADGPASVNQCPV	GTFWYHSHLSTQYCDGLRGAF	HPFHLHGH	GPWFLHCHIDWHLEIGFAMVF
Lac2	HWHGFFQKGTAWADGPAFVTQCPI	GTFWYHSHLSTQYCDGLRGAF	HPFHLHGH	GPWFLHCHIDWHLEAGLAVVF
Lac3	HWHGFFQKGTNWADGPAMVNQCPI	GTFWYHSHLSTQYCDGLRGPF	HPLHLHGH	GPWFLHCHIDWHLEAGFALIF
Lac4	HWHGFFQKGTNWADGPAFVTQCPI	GTFWYHSHLSTQYCDGLRGAF	HPFHLHGH	GPWFLHCHIDWHLEAGFAVVL
Lac5	HWHGFFQAGTNWADGPAFVNQCPI	GTFWYHSHLSTQYCDGLRGAF	HPFHLHGH	GPWFLHCHIDWHLEAGLAVVF
Lac6	HWHGFFQRGSSWADGADSVTQCPI	GTFWYHSHSRTQYCDGLRGAM	HPFHLHGH	GPWFLHCHIDWHLDAGLAIVF
Lac7	HWHGFFQKGTNWADGPAFVNQCPI	GTYWYHSHLSTQYCDGLRGAF	HPFHLHGH	GPWFLHCHIDWHLEAGFAVVF
Lac8	HWHGFFQKGTNWADGPSFVNQCPI	GTYWYHSHLSTQYCDGLRGAF	HPFHLHGH	GPWFLHCHIDWHLEGGLAVVF
		●		● ●

Amino acids (Ser, Glu and Phe) with proposed roles in determining the redox potential are indicated by dark dots underneath.

Table S4. Alignment of the potential substrate-binding loops of *Cerrena* sp. HYB07 laccases.

Protein	Loop I B1-B2	B4-B5	Loop II B7-B8	Loop III C1-C2	Loop IV C4-C5	C7-C8
Lac1	TLARKLKGPPVPD	ISCDPNY	ANPNAG-MKGFDGGIN	FGFANG------------HFT	LAATRKAVGGP	HIDWHLEIGF
Lac2	VLARTVVGVATPD	ISCDPNY	AKPNIGTDTTTTNGMN	LSFAAG------------RFS	IPAG--VVGGP	HIDWHLEAGL
Lac3	TLARLGAAFPTPD	LSCDPNF	ANPNLG-TTGFANGIN	FAFNGSAL----------QFT	MPGG--VVGGG	HIDWHLEAGF
Lac4	ALAQTVVGPAVSD	ISCDPNF	AKPNIG-NTTFLGGLN	FGFSNG------------RFT	LPAAG-AVGGP	HIDWHLEAGF
Lac5	VLAPTVKFTATPD	IGCFPNY	AVPNLG-DKSTDKGIN	LSFSSN------------RFF	IPPR--AHDGP	HIDWHLEAGL
Lac6	TLSPNMSGKPTPD	ISCDPFY	AKPNNARDPSFNGGLN	LSFQNVTDPDTKEVAGKFM	IPPL--KIGGP	HIDWHLDAGL
Lac7	TLARQIVGVAIAD	ISCDPNY	ANPNLG-TTGFTGGIN	LGFSAG------------KFT	LAAG--VLGGP	HIDWHLEAGF
Lac8	TLAHKNVDVPVAD	TSCEPNY	ANPNVG-TPGFAGGIN	VGLTPNGL----------LYT	LAAD--VIAGP	HIDWHLEGGL
	○ ○	○	○	○ ○		○
	●●●	●	●		●●●	● ●

The potential substrate-binding loops were named according to nomenclature of Hakulinen et al. [3] for the two β-strands flanking the respective loop. Open circles underneath indicate the residues corresponding to amino acids in contact with the substrate 2,5-xylidine in LacIIIb of *T. versicolor* [4]. Closed circles underneath indicate the residues corresponding to amino acids in contact with the substrate 2,5-xylidine in Lac7 as proposed by molecular docking (Figure 1B).

Table S5. Predicted secondary structures of *Cerrena* sp. HYB07 laccases.

	α-Helix (%)	Extend Strand (%)	β-Turn (%)	Random Coil (%)
Lac1	7.74	29.01	6.19	57.06
Lac2	11.22	30.23	5.32	53.23
Lac3	11.20	29.34	7.14	52.32
Lac4	10.66	28.68	6.59	54.07
Lac5	12.19	29.71	7.24	50.86
Lac6	7.93	29.52	5.54	57.01
Lac7	10.66	29.46	6.20	53.68
Lac8	11.95	29.48	6.55	52.02

Table S6. geNorm stability ranking (from most stable to least stable) of *Cerrena* sp. HYB07 housekeeping genes during submerged and solid state fermentation.

Rank	Submerged Fermentation	Solid State Fermentation
1	*18S rRNA*	*β-tubulin*
	Cyt-c	*EF1-α*
3	*GAPDH*	*GAPDH*
4	*EF1-α*	*18S rRNA*
5	*β-tubulin*	*ATP6*
6	*ATP6*	*RPB2*
7	*RPB2*	*Cyt-c*

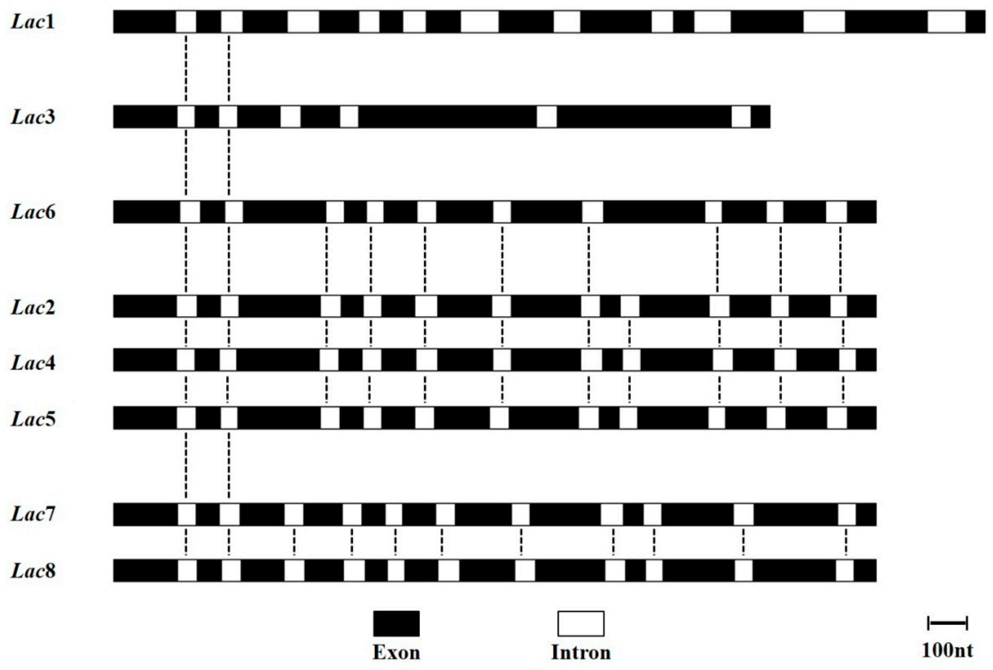

Figure S1. Genetic organization of *Cerrena* sp. HYB07 laccases genes.

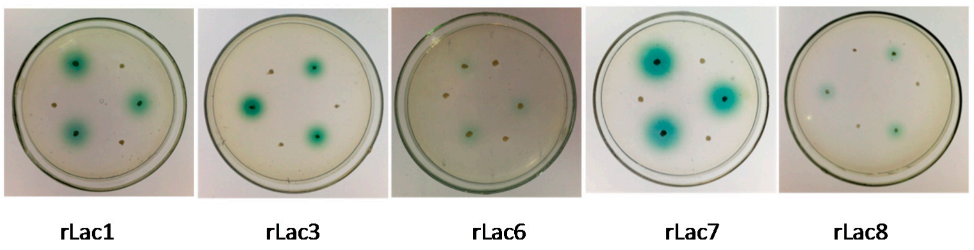

Figure S2. Positive *Pichia* transformants on MM agar plates supplemented with 0.2 mM ABTS.

Colonies of *Pichia* transformants expressing recombinant laccases turned green while negative controls (transformed with pPICZα C) did not.

References

1. D'Souza, T.M.; Boominathan, K.; Reddy, C.A. Isolation of laccase gene-specific sequences from white rot and brown rot fungi by pcr. *Appl. Environ. Microbiol.* **1996**, *62*, 3739–3744.
2. Liu, Z.; Sun, X.; Qu, Y. Cloning cellobiohydrolase I from *penicillium decumbens* 114-2 with tail-pcr and comparing with its derepressed mutant ju-a10. *Acta Microbiol. Sin.* **2008**, *48*, 667–671.
3. Hakulinen, N.; Kiiskinen, L.-L.; Kruus, K.; Saloheimo, M.; Paananen, A.; Koivula, A.; Rouvinen, J. Crystal structure of a laccase from *melanocarpus albomyces* with an intact trinuclear copper site. *Nat. Struct. Biol.* **2002**, *9*, 601–605.
4. Bertrand, T.; Jolivalt, C.; Briozzo, P.; Caminade, E.; Joly, N.; Madzak, C.; Mougin, C. Crystal structure of a four-copper laccase complexed with an arylamine: Insights into substrate recognition and correlation with kinetics. *Biochemistry* **2002**, *41*, 7325–7333.
